# Sacubitril Valsartan Enhances Cardiac Function and Alleviates Myocardial Infarction in Rats through a SUV39H1/SPP1 Axis

**DOI:** 10.1155/2022/5009289

**Published:** 2022-09-22

**Authors:** Jian-Fen Shen, Zhong-Bao Fan, Chun-Wei Wu, Guo-Xian Qi, Qiu-Yu Cao, Feng Xu

**Affiliations:** ^1^Department of Cardiology, The First Hospital of China Medical University, Shenyang, 110001 Liaoning, China; ^2^Department of Hepatobiliary Surgery, People's Hospital of China Medical University, Liaoning Provincial People's Hospital, Shenyang, 110016 Liaoning, China; ^3^Department of Geriatric Cardiology, The First Hospital of China Medical University, Shenyang, 110001 Liaoning, China

## Abstract

Sacubitril valsartan (lcz696) has been demonstrated as a substitute for angiotensin-converting enzyme inhibitors and angiotensin receptor blockers for the treatment of heart failure. This research is aimed at examining the effects of lcz696 and its target molecules on myocardial infarction (MI). A rat model of MI was induced by left anterior descending artery ligation and treated with lcz696. Lcz696 treatment significantly reduced cardiac injury and heart failure, restored the left ventricular fractional shortening and ejection fraction, and reduced oxidative stress and inflammatory responses in rat myocardium. By analyzing the heart failure-related GSE47495 dataset and performing gene ontology (GO) functional enrichment analysis, we obtained histone lysine methyltransferase SUV39H1 and secreted phosphoprotein 1 (SPP1) as two molecules implicated in the oxidative stress and inflammation processes. An elevation of SUV39H1 whereas a decline of SPP1 were detected in cardiac tissues after lcz696 treatment. Enrichments of SUV39H1 and H3K9me3 at the SPP1 promoter were identified by chromatin immunoprecipitation assay. SUV39H1 catalyzed H3K9me3 modification to suppress the expression of SPP1. Preconditioning of SUV39H1 silencing blocked the protective roles of lcz696, but SPP1 silencing alleviated the myocardial injury. In conclusion, this study demonstrates that lcz696 enhances cardiac function and alleviates MI in rats through a SUV39H1/SPP1 axis.

## 1. Introduction

Myocardial infarction (MI), referred to heart attack in lay terms, is usually caused by a decline or stoppage of blood flow to the heart, leading to heart muscle necrosis and cardiac injury due to the insufficient oxygen supply [1]. The formation of blood clot in the epicardial artery is generally involved but not always etiologically necessarily required for MI cases as the myocardial damage can also be induced by an imbalanced blood supply–oxygen demand ratio [2]. Therefore, the current universal definition of MI states that there must be a fall or rise (or both) in a heart muscle damage-sensitive blood test (troponin I or T) with at least one value exceeding the 99^th^ percentile of the upper reference limit [3, 4]. The MI involves several factors, such as ventricular remodeling, recurrent myocardial ischemia, infarct size, stunned myocardium, and mechanical complications [5]. The generation of oxidative stress, inflammation, calcium overload, and cellular apoptosis further deteriorates the situation [6].

The understanding of the etiology, diagnosis, and therapeutic options of acute MI has rapidly evolved over the last 40 years, but great challenges remain [2]. Traditional drugs have presented ideal treatment effect in ameliorating cardiac function [7–9]. Activation of the reninangiotensin-aldosterone system (RAAS) has been recognized to participate in the process of ventricle remodeling and heart failure following MI, leaving angiotensin-converting enzyme inhibitors (ACEIs) and angiotensin receptor blockers (ARBs) which suppress RAAS activity as therapeutic options for heart failure [10]. Sacubitril valsartan (lcz696), a so-called angiotensin receptor—neprilysin inhibitor, is comprised of the neprilysin inhibitor sacubitril and the ARB valsartan [11]. Lcz696 has been approved for the treatment of heart failure with reduced ejection fraction (HFrEF) and used as substitute for ARBs and ACEIs [12, 13]. In addition to its long-term benefits on cardiac function, lcz696 recently has been demonstrated to potentially alleviate cardiac dysfunction in acute MI [14, 15]. However, the roles of lcz696 in cardiac function, oxidative stress, and inflammation following MI, especially the molecules it affected are not fully understand.

Advanced bioinformatics tools have offered great convenience to the prediction and identification of hub genes in human diseases, including MI [16]. In the present study, by analyzing the heart failure-related GEO dataset GSE47495 (https://www.ncbi.nlm.nih.gov/geo/query/acc.cgi?acc=GSE47495) and performing gene ontology (GO) functional enrichment analysis, we obtained histone lysine methyltransferase SUV39H1 and secreted phosphoprotein 1 (SPP1) as two molecules implicated in the oxidative stress and inflammation processes during MI. SUV39H1 is a mammalian lysine methyltransferase which modulates di- and tri-methylation of histone 3 lysine 9 (H3K9me2/3) [17]. SUV39H1 defect leads to significant H3K9me3 deduction and SUV39H1 results in facultative heterochromatin formation and gene silencing by promoting H3K9me3 modification [18]. Upregulation of SUV39H1 has been reported to reduce infarct size and tissue damage following myocardial ischemia-reperfusion injury [19]. Moreover, SPP1 has also been documented as one of the hub genes in MI [16]. Taken together, this study was launched to explore the exact function of lcz696 in MI and the potential involvements of SUV39H1 and SPP1.

## 2. Materials and Methods

### 2.1. Animals

Mature male SD rats (6 weeks old, 180-220 g) procured from SPF (Beijing) Biotechnology Co., Ltd. (Beijing, China) were used for in vivo experiments. The rats were separately housed in standard conditions at room temperature (22-25°C) in a 12 : 12 h dark/light cycle. The animal usage was approved by the Animal Ethics Committee of the First Hospital of China Medical University (Approval No. CMU20210305), and all procedures were abided by the Guide for the Care and Use of Laboratory Animals (NIH, Bethesda, Maryland, USA).

### 2.2. MI in Rats Induced by Ligation of the Left Anterior Descending (LAD) Artery

After one week of adaptation, the rats were fixed on the table with an integrated biological signal acquisition and processing system (BL-422I; Techman Co., Ltd., Chengdu, Sichuan, China) in the laboratory. The rats were anesthetized via intraperitoneal injection of 1% pentobarbital sodium (50 mg/kg) and connected to an electrocardiograph. After deep anesthesia, the rats were shaved, disinfected, and the neck skin was incised. The fascias were separated layer by layer to expose the trachea. An inverted Y-shape incision was made in the 3-4 cartilage space of trachea, and a tracheal device was instantly inserted when the connected ventilator was turned on. Thereafter, the skin was incised between the 3^rd^ and 4^th^ ribs at the left edge of the sternum. The anadesma and muscles were separated, and the 3^rd^ and 4^th^ ribs were cut off to fully expose the heart. The pericardium was cut open, and the left coronary vein between the conus arteriosus and the root of the left atrial appendage was found. Thereafter, a ligation of the LAD artery was made between 3-4 cm below the left atrial appendage and the conus arteriosus to induce acute MI. For sham operation, the rats received similar procedures except for the ligation of LAD artery. After ligation, the ST-segments of the limb lead and the V1 lead showed a convex-upward elevation. In the ischemic area at the ligation site, the color of myocardium changed from red to dark gray and even to pale white. The rats were observed for 30 min. The successful induction of MI was confirmed by the appearance of pale anterior wall at the left ventricle and an over 0.15 mV ST-segment elevation or depression. After the surgery, the chest was closed after no bleeding was found. The pleural effusion was extracted using a syringe during the suturing. During the postoperation recovery period, the MI rats were treated with the opioids (buprenorphine; 0.1 mg/kg, PO) for analgesia.

### 2.3. Plasmids and Drug Treatments

Small interfering RNA (siRNA) of SUV39H1 (si-SUV39H1) and SPP1 (si-SPP1) and the empty plasmids (negative control; NC) were chemical-modified and designed by the Sangon Biotech Co., Ltd. (Shanghai, China). Each heart was injected with 50 *μ*g plasmid dissolved in 50 *μ*L RNase-free water using a 30-gauge needle and a 10 *μ*L Hamilton injector. Five injections were performed (10 *μ*L/per injection), of which three injections were performed at the border site of the infarcted area and two injections at the center of the infarcted area. One week later, the rats were given 68 mg/kg lcz696 (bioavailable oral formulations composed of valsartan and sacubitril in a molar ratio of 1 : 1; Selleck Chemicals, Houston, TX, USA) orally a day for continuous seven weeks. MI rats treated with equal doses of solvent (DMSO) were set to controls.

### 2.4. Hemodynamics

Rats in each group were injected with 1,200 U/kg heparin sodium for 20 min of anticoagulation. Thereafter, the rats were anesthetized via inhalation of 2% isoflurane (0.41 mL/min at 4 L/min fresh gas flow). A tracheal cannula was inserted into the right common carotid artery, and a PE-50 polyethylene catheter (ICU Medical Inc., San Clemente, CA, USA) connected to a high-precision pressure transducer was inserted. The hemodynamic parameters including systolic blood pressure (SBP), diastolic blood pressure (DBP), and mean arterial pressure (MAP) were measured using a PowerLab biological signal processing and analyzing system (ADInstruments Ltd., Sydney, NSW, Australia). The catheter was further inserted into the left ventricle after 10 min of stabilization in the carotid artery. After 5 min, the left ventricular (LV) systolic pressure (LVSP), LV end-diastolic pressure (LVEDP), and the maximum rate of change in LV pressure (± LVdp/dtmax) were recorded.

### 2.5. Echocardiography

Under anesthesia by 2% isoflurane, the rats were subjected to echocardiography using a 12.0 MHz transducer-attached Vivid E9 diagnostic ultrasound system (General Electric Co., NY, USA). The two-dimensional and M-mode echocardiographic images were obtained in parasternal long axis and short axis views of the heart. All measurements were performed online, with the best images from >10 cardiac cycles taken by an experienced ultrasound physician who was unaware of the study protocol and grouping. In the parasternal short axis image at the papillary muscle level, the LV end-diastolic diameter (LVEDD) and LV end-systolic diameter (LVESD) were measured using the M-mode. Thereafter, the LV fractional shortening (LVFS) was calculated as follows: LVFS (%) = (LVEDD − LVESD)/LVEDD × 100. The LV ejection fraction (LVEF) was calculated according to the Teichholz formula [20]. The total heart weight (HW) and the total weight of left ventricle (LV) and right ventricle (RV) in the resected heart was normalized to the tibia length (TL) to obtain the values of HW/TL and LV+RV/TL.

### 2.6. Masson's Trichrome Staining

After the echocardiography measurements, the rats were euthanized by intraperitoneal injection of overdosed pentobarbital sodium (150 mg/kg). The separated cardiac tissue samples were fixed in 4% paraformaldehyde (PFA) and embedded in paraffin to prepare 5 *μ*M sections. The nuclei were stained with Wiegert hematoxylin solution (Sigma-Aldrich, Merck KGaA, Darmstadt, Germany) for 5 min. The tissue sections were stained with 0.7% acid fuchsin (Sigma-Aldrich) for 10 min, washed with 2% glacial acetic acid, classified with phosphomolybdic acid for 4 min, stained with 2% aniline blue (Sigma-Aldrich), treated with graded ethanol and xylene, and sealed with neutral resin. Thereafter, the staining was observed under an optical microscope (Zeiss, Germany), and the infarct area (collagen deposition) and the endocardium perimeter were examined using the ImageJ software by three pathologists blind to the grouping details. For infarct area analysis, the infarct area (blue) was determined by the set threshold color (threshold color: 127~197), and its percentage in total area (threshold color: 0 ~ 255) was calculated. For the percentage of infarcted endocardium, the blue infarct area (threshold color: 127~197) was selected. The endocardium was labeled and its length in the infarct area was measured by the “polygon selections” of ImageJ, and its percentage in the total endocardial perimeter was then calculated.

### 2.7. Hematoxylin and Eosin (HE) Staining

The rat cardiac tissues were fixed in 4% PFA for 24 h and cut into sections. The sections of the infarct border zone were dewaxed, rehydrated in graded alcohol for 5 min, and stained with hematoxylin solution (Solarbio) for 5 min. Thereafter, the sections were differentiated in 1% hydrochloric acid-ethanol for 3 s and stained with 5% eosin solution (Solarbio) for 3 min. After dehydration, the sections were sealed by neutral balsam and observed under the inverted microscope with five random fields included. The cardiac damage was scored by three pathologists from two aspects including inflammatory infiltration and cardiomyocyte morphology. The inflammatory infiltration was scored as follows: 0, no significant infiltration; 1, mild infiltration; and 2, severe infiltration. The cardiomyocyte morphology change was scored as follows: 0, normal structure of cells; 1, a small number of necrotic cardiomyocytes with disordered structure and fibrosis progression; and 2, a large number of necrotic cardiomyocytes with disordered structure and fibrosis progression. The final score was determined by the sum of the two separate scores (0 ~ 4).

### 2.8. Examination of Oxidative Stress-Related Factors

Total concentration of ROS (Cat. No. S0033S), total glutathione (GSH; Cat. No. S0052), the activities of total superoxide dismutase (SOD; Cat. No. S0109), and glutathione peroxidase (GPx; Cat. No. S0056) in infarct border zone tissues were examined using the corresponding colorimetric assay kits (Beyotime Biotechnology Co., Ltd., Shanghai, China) in accordance with the manufacturer's protocols.

### 2.9. Examination of Inflammatory Cytokines

The homogenate of infarct border zone tissues was prepared on ice and centrifuged at 3,000 rpm for 10 min to collect the supernatant. The cardiomyocytes were collected and washed in phosphate-buffered saline (PBS), centrifuged at 4°C for 10 min, and then at 12,000 rpm for 5 min to collect the supernatant. The levels of inflammatory cytokines including tumor necrosis factor-*α* (TNF-*α*; CSB-E11987r, Cusabio Technology LCC, Houston, TX, USA), interleukin (IL)-6 (CSB-E04640r, Cusabio Technology) and IL-1*β* (E-EL-R0012c, Elabscience Biotechnology Co., Ltd., Wuhan, Hubei, China), and the concentration of the anti-inflammatory IL-10 (E-EL-R0016c, Elabscience) in the supernatant samples were examined using enzyme-linked immunosorbent assay (ELISA) kits according to the manufacturer's protocols.

### 2.10. Immunohistochemistry (IHC)

Paraffin-embedded tissue sections were dewaxed and rehydrated for IHC assay. The sections were water-bathed in antigen retrieval solution (Solarbio) for 1 h, blocked with normal goat serum (Solarbio) at 23°C for 20 min, and then incubated with anti-Ki67 (1 : 5,000, ab279653, Abcam Inc., Cambridge, MA, USA), anti-SUV39H1 (1 : 800, MA1-25505, Thermo Fisher Scientific), and anti-SPP1 (1 : 5,000, NB110-89062, Novus Biologicals, Littleton, CO, USA) at 4°C overnight, and then incubated with the goat antimouse IgG (1 : 1,000, ab205719, Abcam). Thereafter, the sections were cultured with horseradish peroxidase- (HRP-) labeled streptavidin (Solarbio) at 37°C for 20 min, developed with DAB (Solarbio), and counter-stained with hematoxylin (Solarbio) for 1 min. After that, the tissue sections were dehydrated, cleared in xylene, and sealed with neutral resin. The number of IHC-positive cells (brownish) was counted under the microscope. The rate of positive cells was calculated by three pathologists blind to the groups as follows: rate = positive cells/total cells × 100%.

### 2.11. Terminal Deoxynucleotidyl Transferase- (TdT-) Mediated dUTP Nick End Labeling (TUNEL)

Cell apoptosis in cardiac tissues (the infarct border zone) was examined using an ApopTag® Fluorescein In Situ Apoptosis Detection Kit (Merck KGaA) and a fluorescence microscope (Zeiss). The 5 *μ*M sections were rehydrated and incubated with proteinase K (Invitrogen; Thermo Fisher Scientific Inc., Waltham, MA, USA) at 25°C for 30 min. The sections were warm-incubated with the TUNEL reaction mixture at 37°C in the dark and humidified condition for 60 min. For cultured cardiomyocytes, the cells were incubated with TUNEL reagent mixture for 30 min. After that, the nuclei were stained with DAPI in the dark for 30 min. The labeling was observed under the microscope. The percentage of apoptotic cells in total cells in tissues was calculated by three pathologists blind to the groups using the ImageJ software (NIH).

### 2.12. Cell Culture and Treatment

H9C2 cardiomyocytes (China Center for Type Culture Collection, Wuhan, Hubei, China) were cultured in a humidified incubator at 37°C with 5% CO_2_. The cells were cultured in DMEM (Thermo Fisher Scientific) supplemented with 10% fetal bovine serum, 100 U/mL penicillin and 100 *μ*g/mL streptomycin. The cells were digested in 0.25% trypsin and 0.02% EDTA. Cells at passage three were collected for subsequent use.

The siRNA of SUV36H1 and SPP1 or the NC were transfected into H9C2 cells following the instruction manual of Lipofectamine 2000 (Thermo Fisher Scientific). In short, 1.25 *μ*L siRNA storage solution (20 *μ*M) or 1 *μ*L Lipo2000 reagent was diluted in 50 *μ*L serum-free Opti-MEM. The two dilutions were allowed to stand at room temperature for 5 min and then mixed for 20 min to form siRNA-Lipo2000 mixture. The mixture was then loaded in H9C2 cells in 24-well plates (1 × 10^5^ cells/well) with 400 *μ*L culture solution. The final concentration of the siRNA was 50 nM. After 4 ~ 6 h, the culture medium was replaced by fresh medium without transfection reagent. After 48 h of incubation, the transfection efficiency was determined.

### 2.13. Cell Counting Kit-8 (CCK-8) Method

After 48 h, cell viability was determined using a CCK-8 kit (Beyotime). Each well was filled with 10 *μ*L CCK-8 solution followed by 2 h of incubation at 37°C. The optical density (OD) value at 450 nM was evaluated using a microplate reader (Epoch; BioTek Instruments, Shanghai, China).

### 2.14. Flow Cytometry

The H9C2 cells were digested in 0.25% trypsin. Apoptosis of cells was determined using an annexin V-fluorescein isothiocyanate (FITC)/propidium iodide (PI) kit (Beyotime). The cells were resuspended in 1× binding buffer and then incubated with 5 *μ*L annexin V-FITC and 10 *μ*L PI at 37°C in the dark for 15 min. The apoptotic cells were analyzed using a FACSCalibur flow cytometer (BD Biosciences) and the Cell Quest Software (v.3.3; BD Biosciences). The numbers of both early-apoptotic cells (annexin V-FITC positive) and late apoptotic cells (PI positive) were calculated.

### 2.15. Bioinformatics Analysis

The GEO dataset GSE47495 containing gene expression profiling (by array) in LV and peripheral blood mononuclear cells of MI rats was downloaded for gene differential expression analysis. The data in the dataset were obtained from sham-operated rats (*n* = 6), and rats with low (*n* = 6), medium (*n* = 6), and high (*n* = 5) grades MI at two months after infarction examination. The LV and blood samples were used for RNA extraction and hybridization in the Affymetrix microarray. In the present study, only the gene expression data in the LV sample of the sham-operated rats and the high-grade MI rats were included for analysis. The dataset was loaded into the edgeR package (Bioconductor, Seattle, WA, USA). Differentially expressed genes (DEGs) were identified using |fold change| > 1 and *p* value <0.01 as the screening thresholds. The volcano plots were produced using R Package ggplot2 (NIH). A GO functional enrichment analysis was performed in the DAVID system (https://david.ncifcrf.gov/summary.jsp) to identify the biofunctional processes the DEGs enriched in. The Sankey dot pathway enrichment plots of the analysis results were generated using the R Package Sankey D3. The histone modification peaks at the SPP1 promoter in LV were obtained from ENSEMBL (http://asia.ensembl.org/).

### 2.16. Reverse Transcription-Quantitative Polymerase Chain Reaction (RT-qPCR)

Total RNA from the cardiac tissues (infarct border zone) was extracted using the TRIzol Reagent (Thermo Fisher Scientific). The RNA concentration was examined by ultraviolet analysis, and the integrity was examined by electrophoresis. The total RNA was reverse-transcribed to the first-strand cDNA using a PrimeScript RT with gDNA Eraser (Perfect Real Time) (Takara Holdings Inc., Kyoto, Japan). Thereafter, qPCR was conducted using a TB Green® Premix Ex Taq™ II (Tli RNase H Plus; Takara) on a LightCycler 480 real-time PCR system (Roche Diagnostics Ltd., Risch, Switzerland). GAPDH was used as the internal control for mRNA. Relative gene expression was examined by the 2^−ΔΔCt^ method. The primers are listed in Table 1.

### 2.17. Western Blot Analysis

The homogenate of infarct border zone tissue was lysed in RIPA lysis buffer (Beyotime) and centrifuged at 4°C at 1,000 × *g* for 10 min to collect total protein. After protein concentration examination with a bicinchoninic acid assay kit (Beyotime), an equal amount of protein sample (30 *μ*g) was separated by 10% SDS-PAGE and loaded onto PVDF membranes (Millipore Corp., Billerica, MA, USA). After treatment with 5% nonfat milk for 1 h, the membranes were hybridized with the following primary antibodies at 4°C overnight: anti-SUV39H1 (1 : 1,500, ab12405, Abcam), anti-SPP1 (1 : 2,000, NB110-89062, NOVUS Biologicals), anti-H3K9me3 (1 : 1,000, ab6721, Abcam), anti-H3 (1 : 1,300, ab1791, Abcam), and anti-GAPDH (1 : 1,500, ab9485, Abcam). After that, the membranes were washed and hybridized with goat antirabbit IgG (1 : 1,000, ab205718, Abcam) or goat antimouse IgG (1 : 1,000, ab205719, Abcam) at 23°C for 1 h. The protein bands were visualized using an electrochemiluminescence kit (Pierce, Thermo Fisher Scientific) and exposed to X-ray film. GAPDH was used as the endogenous loading.

### 2.18. Chromatin Immunoprecipitation (ChIP)-qPCR

The ChIP assay was conducted using a SimpleChIP Plus Enzymatic Chromatin IP kit (Cell Signaling Technology [CST], Beverly, MA, USA). The H9C2 cells were crosslinked in 1% methanol for 10 min and terminated by glycine. The cells were then scraped off, and the nuclei were separated and lysed. After ultrasonication, the chromatin part was separated. The chromatin extract was incubated with anti-SUV39H1 (1 : 500, ab12405, Abcam) or the control IgG (CST) at 4°C overnight for IP reaction. The DNA-protein complexes were decrosslinked, and the expression of target DNA was quantified by qPCR analysis.

### 2.19. Statistical Analysis

SPSS22.0 was applied for data analysis (IBM Corp., Armonk, NY, USA). Measurement data were presented as the mean ± standard deviation. Three independent experiments were performed. The intergroup difference was compared by the unpaired *t*-test, or by the one- or two-way analysis of variance (ANOVA) followed by Tukey's post-hoc test. *p* < 0.05 was set as the cut-off value for significant difference.

## 3. Results

### 3.1. Physiological Changes in Rats during LAD Artery Ligation

**Figure 1:**
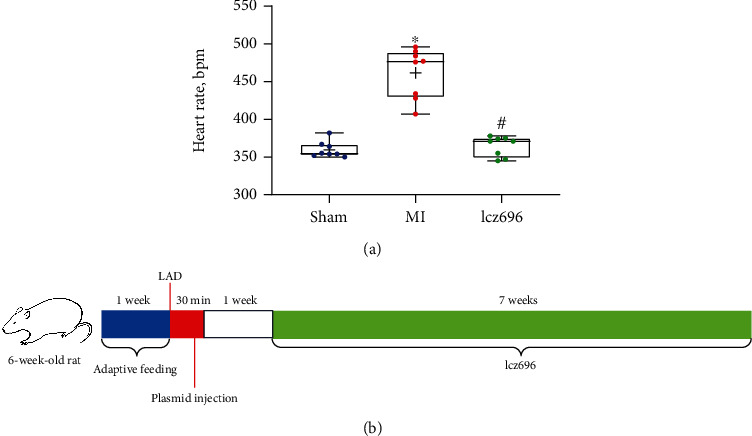
Physiological changes in rats during LAD artery ligation. (a) Heart rate of rats in each group. (b) A flow diagram for the treatment of rats. The rats were sacrificed at week 9 via intraperitoneal injection of 150 mg/kg pentobarbital sodium. The sham group refers to rats underwent sham operation without LAD (left anterior descending) artery ligation; the MI (myocardial infarction) group refers to the model group where rats induced with MI via LAD artery ligation; and the lcz696 group refers to rats with MI treated with lcz696. Differences were analyzed by the one-way ANOVA. ^∗^*p* < 0.05 vs. the sham group; ^#^*p* < 0.05 vs. the MI group.

The rats were fixed on the table with a biological experimental system in the laboratory. They were anesthetized and equipped with an electrocardiograph connecting the limb lead and the V1 lead. A tracheal device connected to a ventilator was inserted to the rat trachea. The ventilator was turned on in the following settings: respiratory rate, 75 times/min; tidal volume, 3 mL/100 g; and inspiratory-to-expiratory ratio, 1 : 3. The MI in rats was induced by ligation of the LAD artery. After chest closure, the physiological status of rats under ventilator support was observed. The rat tail was clipped using a forcep, and the tongue tip was stimulated with a wet cotton swab. When the rats responded to the stimulations, the ventilator support was suspended. The abdominal respiratory status was observed. If the rats regained spontaneous breathing, the trachea was removed. Animal anesthesia was precisely controlled under strict supervision by highly qualified experimenters, and low rates of intragroup variability was observed throughout the period (Figure 1(a)). The plasmids were intramyocardially injected into rats 30 min after LAD ligation. One week later, the rats were given lcz696 orally once a day for continuous seven weeks (Figure 1(b)). After MI induction, the rats showed different degrees of drooping spirit, reduced activity, lagging skin, lack of gloss, poor diet, and shortness of breath. Several autopsies showed pleural and peritoneal effusions. Rats underwent LAD artery ligation were assigned into the following groups: MI group, lcz696 group, si-NC group, si-SUV39H1 group, and si-SUV39H1+si-SPP1 group. At first, each group contained 8 rats. When animals died, new model rats were added to maintain a total of 8 successfully modeled live rats in each group during the 8-week experiment period. In the MI group, 2 rats died from pneumothorax and 2 rats died from heart failure during the whole process. One rat died from infection in the lcz696 and si-NC groups, respectively. In the si-SUV39H1 group, 1 rat died from infection, 2 rats died from heart failure, and 1 died from sepsis. In the si-SUV39H1+si-SPP1 group, 1 rat died from infection and 1 from heart failure. The details of animal death in each group are shown in Table 2. The hemodynamics analysis showed that the levels of SBP, DBP, and MAP were reduced in the MI rats but restored after lcz696 treatment (Table 3). Moreover, the LV dynamics showed that the model rats showed declined LVSP, elevated LVEDP, and reduced ± LVdp/dtmax. Treatment of lcz696 elevated the LVSP, reduced the LVEDP, and increased ± LVdp/dtmax of rats with MI (Table 4).

### 3.2. Lcz696 Treatment Improves Cardiac Function of MI Rats

**Figure 2:**
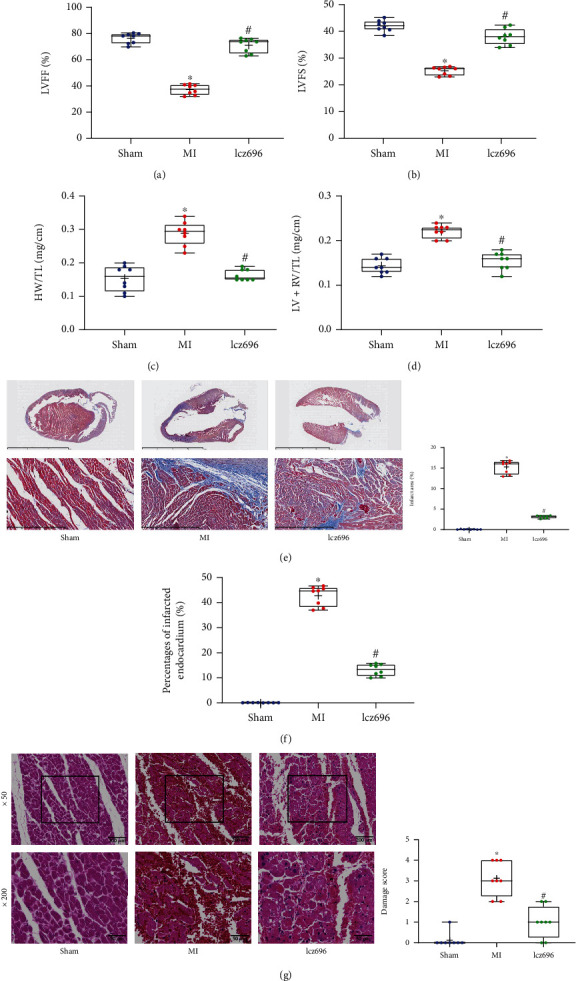
Lcz696 treatment improves cardiac function of MI rats. (a, b) LVEF (a) and LVFS (b) values of MI rats as manifestations of myocardial function (*n* = 8). (c, d) The cardiac remodeling in rats examined by HW/TL (c) and LV+RV/TL (d) values (*n* = 8). (e) Infarct area in the cross section of LV examined by Masson's trichrome staining (*n* = 8). (f) Percentage of the perimeter of infarcted endocardium in total endocardium (*n* = 8). (g) Pathological changes in rat myocardium determined by HE staining (*n* = 8). The sham group refers to rats underwent sham operation without LAD (left anterior descending) artery ligation; the MI (myocardial infarction) group refers to the model group where rats induced with MI via LAD artery ligation; and the lcz69l group refers to rats with MI treated with lcz696. Differences were analyzed by the one-way ANOVA. ^∗^*p* < 0.05 vs. the sham group; ^#^*p* < 0.05 vs. the MI group.

According to the two-dimensional echocardiographic images, the LVEF of MI rats was declined from the baseline value 75 ± 1% to 37 ± 1%. The lcz696 treatment significantly elevated the LVEF of rats, and the elevation was greater on week 5 (Figure 2(a)). Moreover, the echocardiographic analysis showed that the LVFS of MI rats was declined but enhanced after lcz696 treatment as well (Figure 2(b)). The weight of heart normalized to TL was calculated. Significant LV remodeling was found in MI rats, as manifested by increased HW/TL and LV+RV/TL values compared to the sham-operated rats. Of note, the HW/TL and LV+RV/TL values were reduced by lcz696 treatment (Figures 2(c) and 2(d)). Masson's trichrome staining was also performed to examine the infarct area in the cross section through the mid LV. Likewise, increased infarct size was detected in MI rats, whereas reduced infarct size was observed in rats following lcz696 treatment (Figure 2(e)). The percentage of the perimeter of the infarcted endocardium was examined as well. It was observed that the endocardial infarction was increased in MI rats but reduced by lcz696 treatment (Figure 2(f)). HE staining was performed to examine the pathological changes in rat cardiac tissues. In the sham group, the tissue was evenly stained, and there was no significant inflammatory infiltration; the myocardial cells had normal structure, the muscle fibers were neatly arranged with no obvious pathological changes. However, in the MI group, aggravated myocardial injury and increased myocardial interstitial edema were observed. The lcz696 treatment alleviated the pathological changes (Figure 2(g)).

### 3.3. Lcz696 Treatment Reduces Oxidative Stress and Inflammation in Rat Myocardium

**Figure 3:**
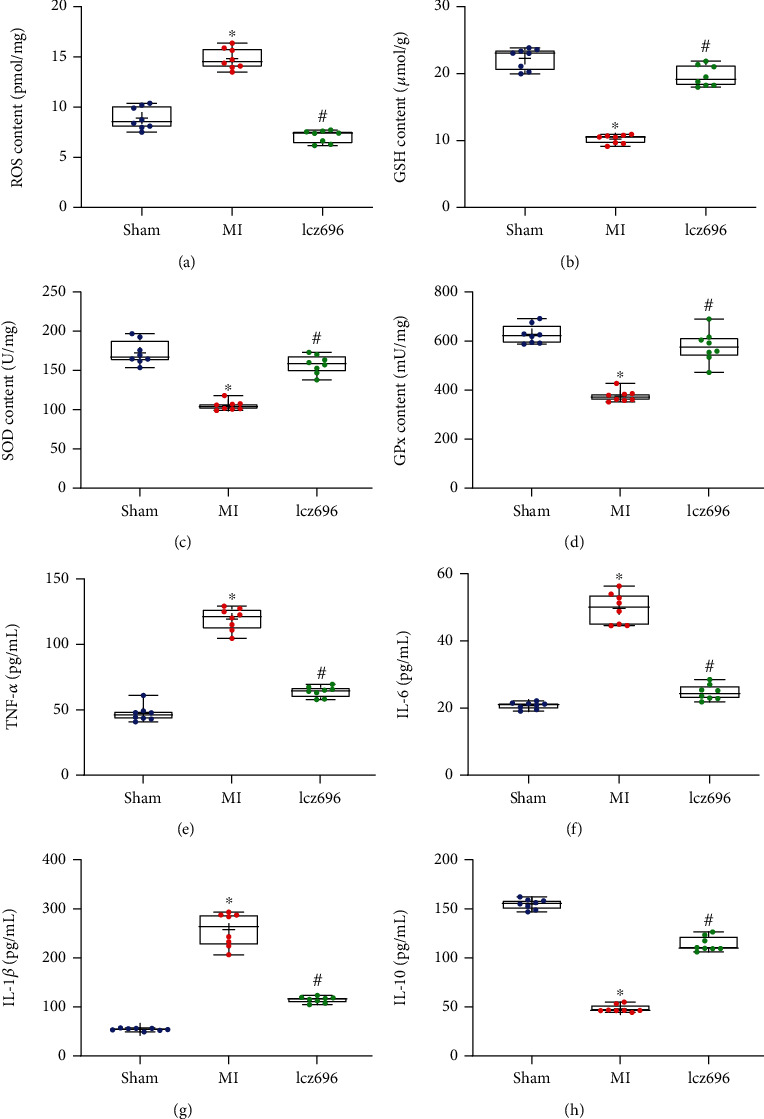
Lcz696 treatment reduces oxidative stress and inflammatory response in rat myocardium. (a–d) Concentration of oxidative stress-related cytokines in rat myocardium examined by the colorimetry. (e–h) Production of proinflammatory cytokines TNF-*α* (e), IL-6 (f), and IL-1*β* (g) and the anti-inflammatory IL-10 (h) in rat myocardium examined by using ELISA kits (*n* = 8). The sham group refers to rats underwent sham operation without LAD (left anterior descending) artery ligation (*n* = 8); the MI (myocardial infarction) group refers to the model group where rats induced with MI via LAD artery ligation; and the lcz69l group refers to rats with MI treated with lcz696. Differences were analyzed by the one-way ANOVA. ^∗^*p* < 0.05 vs. the sham group; ^#^*p* < 0.05 vs. the MI group.

To better understanding the phenotypic difference, we further explored the function of lcz696 in the oxidative stress and inflammation in rat myocardium. As shown in Figure 3(a), the ROS level was increased in the myocardium of MI rats but reduced following the treatment of lcz696. Moreover, the production of GSH in MI rats was significantly reduced in MI but elevated by lcz696 (Figure 3(b)). The lcz696 treatment also restored the concentrations of SOD and GPx that were initially reduced in rat myocardium after MI induction (Figures 3(c) and 3(d)). In terms of inflammation, the MI rats had increased levels of TNF-*α*, IL-6, and IL-1*β* compared to the sham-operated rats, but lcz696 reduced the production of these proinflammatory cytokines in the cardiac tissues (Figures 3(e)–3(g)). In addition, lcz696 treatment induced the release of anti-inflammatory IL-10 in rat myocardium (Figure 3(h)). These results indicate that the lcz696 treatment reduces oxidative stress and inflammatory response in rat myocardium to alleviate MI in rats.

### 3.4. Lcz696 Treatment Reduces Cardiomyocyte Apoptosis in Rat Cardiac Tissues

**Figure 4:**
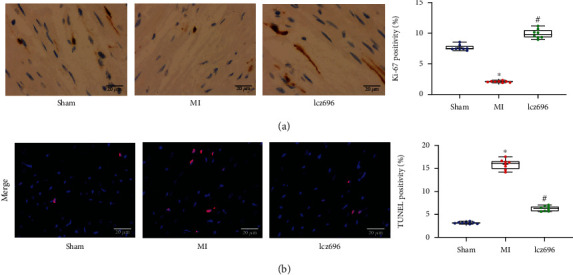
Lcz696 treatment reduces cardiomyocyte apoptosis in rat cardiac tissues. (a) Expression of the proliferation marker Ki-67 in rat cardiac tissues examined by the IHC assay (*n* = 8). (b) Apoptosis of cardiomyocytes in rat cardiac tissues determined by the TUNEL assay (*n* = 8). The sham group refers to rats underwent sham operation without LAD (left anterior descending) artery ligation; the MI (myocardial infarction) group refers to the model group where rats induced with MI via LAD artery ligation; and the lcz69l group refers to rats with MI treated with lcz696. ^∗^*p* < 0.05 vs. the sham group; ^#^*p* < 0.05 vs. the MI group/DMSO group.

The function of lcz696 in the viability of cardiomyocytes in rat cardiac tissues was examined. First, the expression of the proliferation marker Ki-67 in the tissues was examined, which was found to be reduced in the MI rats but recovered after lcz696 treatment (Figure 4(a)). The subsequent TUNEL assay showed that the cell apoptosis in rat cardiac tissues was elevated after MI induction, but this elevation was suppressed by lcz696 treatment (Figure 4(b)).

### 3.5. Lcz696 Regulates the SUV39H1/SPP1 Axis in MI Rats

**Figure 5:**
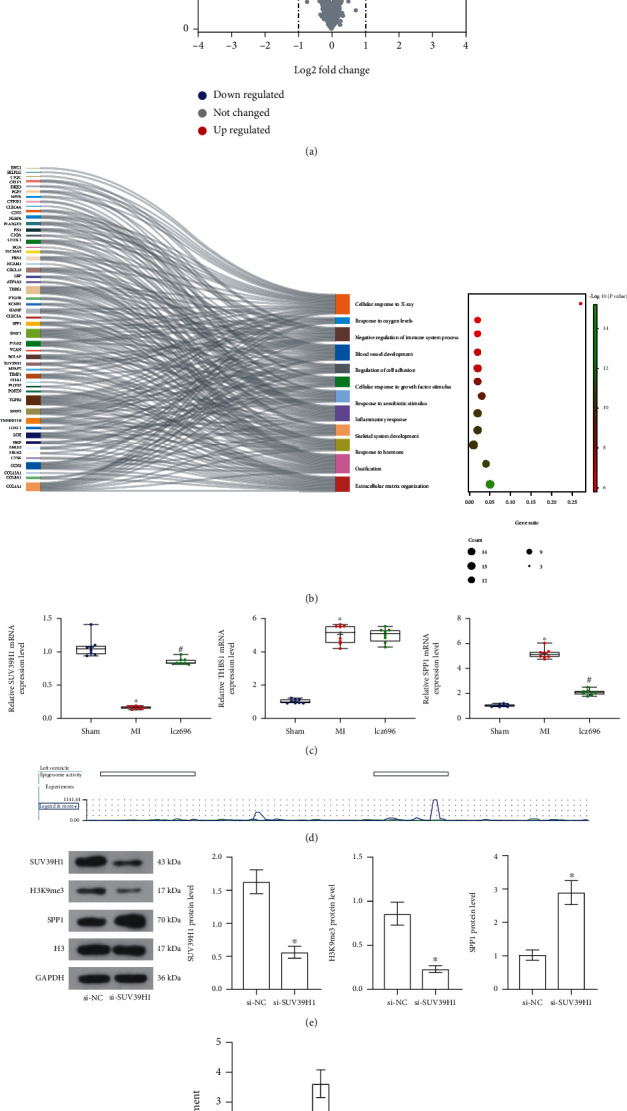
Lcz696 regulates the SUV39H1/SPP1 axis in MI rats. (a) Volcano plots for DEGs in MI rats in the GSE47495 dataset. (b) Biofunctional processes the DEGs-enriched analyzed by GO functional enrichment analysis. (c) mRNA expression of SUV39H1, SPP1, and THBS1 in rat cardiac tissues examined by RT-qPCR (*n* = 8). (d) Histone modification peaks at the SPP1 promoter in LV obtained from the ENSEMBL system. (e) Protein levels of SUV39H1, H3K9me3, and SPP1 in H9C2 cells determined by Western blot analysis (*n* = 3). (f) Enrichment of SUV39H1 and H3K9me3 fragments in SPP1 promoter examined by the ChIP-qPCR (*n* = 3). Differences were analyzed by the unpaired *t*-test and one-way ANOVA or two-way ANOVA. The sham group refers to rats underwent sham operation without LAD (left anterior descending) artery ligation; the MI (myocardial infarction) group refers to the model group where rats induced with MI via LAD artery ligation; the lcz69l group refers to rats with MI treated with lcz696; and the si-NC (small interfering RNA-negative control) and the si-SUV39H1 groups refer to the H9C2 cells transfected with si-NC or si-SUV39H1. ^∗^*p* < 0.05 vs. the sham group/control group/si-NC group; ^#^*p* < 0.05 vs. the MI group.

The molecules involved in the events above were explored. A heart failure-related GSE47495 dataset (expression profiling by array) was analyzed. The results showed that 63 genes were upregulated whereas three genes were downregulated in the cardiac tissues of rat with MI (Figure 5(a)) (Supplementary Table 1). Thereafter, the biofunctional processes the DEGs enriched were analyzed by the GO enrichment analysis. Most of the genes were enriched in the process of myocardial fibrosis, which is right the most important phenotypic change following MI. In addition, several genes were enriched in the oxidative stress and inflammatory response processes, including SUV39H1, SPP1, and thrombospondin 1 (THBS1), indicating that these molecules may function as the key factors participating the oxidative stress and inflammation processes in MI (Figure 5(b)). The expression of SUV39H1, SPP1, and THBS1 in the tissues was examined by RT-qPCR. The SPP1 and THBS1 expression was upregulated and SUV39H1 was downregulated in the rat cardiac tissues after MI induction. In model rats, the lcz696 treatment elevated the level of SUV39H1 and reduced the level of SPP1; however, it did not affect the expression of THBS1 in rat myocardium (Figure 5(c)). As SUV39H1 is an epigenetic regulator which enhances H3K9me3 level to suppress gene expression, we therefore explored if there is a regulation relationship between SUV39H1 and SPP1. The bioinformatics prediction showed that there are H3K9me3 modification peaks at the SPP1 promoter in LV (Figure 5(d)). The expression of SUV39H1 and SPP1 in H9C2 cells was examined to validate the possible interaction between SUV39H1 and SPP1. Thereafter, si-SUV39H1 was transfected into H9C2 cells, after which reduced levels of the SUV39H1 and H3K9me3 whereas increased level of SPP1 were detected (Figure 5(e)). Moreover, the ChIP-qPCR assay suggested that downregulation of SUV39H1 reduced the abundance of SUV39H1 and H3K9me3 fragments enriched by SPP1 (Figure 5(f)). These results indicated that SUV39H1 might modulate H3K9me3 level to suppress SPP1 transcription in MI.

### 3.6. The SUV39H1/SPP1 Axis Modulates Cardiac Function and Myocardial Function in Rats

**Figure 6:**
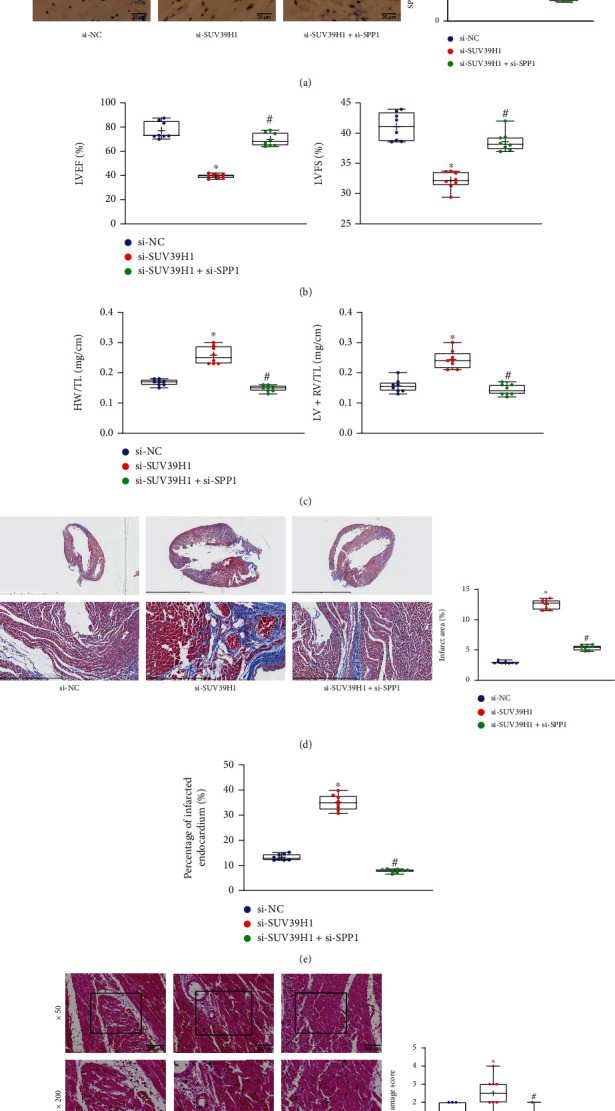
The SUV39H1/SPP1 axis modulates cardiac function and myocardial function in rats. (a) Protein levels of SUV39H1 and SPP1 in rat myocardial tissues examined by the IHC assay (*n* = 8). (b) LVEF and LVFS values of MI rats as manifestations of myocardial function (*n* = 8). (c) The cardiac remodeling in rats examined by HW/TL and LV+RV/TL values (*n* = 8). (d) Infarct area in the cross section of LV examined by Masson's trichrome staining (*n* = 8). (e) Percentage of the perimeter of infarcted endocardium in total endocardium (*n* = 8). (f) Pathological changes in rat myocardium determined by HE staining (*n* = 8). The si-NC (small interfering RNA-negative control) and the si-SUV39H1 groups refer to the H9C2 cells transfected with si-NC or si-SUV39H1. Differences were analyzed by the one-way ANOVA. ^∗^*p* < 0.05 vs. the si-NC group; ^#^*p* < 0.05 vs. the si-SUV39H1 group.

SUV39H1 silencing alone, or the concomitant downregulation of SUV39H1 and SPP1 was introduced in rats, followed by lcz696 treatment. It was found that si-SUV39H1 reduced the level of SUV39H1 but increased the level of SPP1, and si-SPP1 reduced the level of SPP1 in rat myocardium (Figure 6(a)). Moreover, the LVEF and LVFS values of the MI rats were significantly reduced after SUV39H1 silencing but restored after further SPP1 knockdown (Figure 6(b)). The HW/TL and LV+RV/TL values of rats were significantly elevated, namely, the cardiac remodeling was aggravated after SUV39H1 silencing. However, this process was alleviated after SPP1 silencing (Figure 6(c)). The Masson's trichrome staining showed that the infarct area in rat cardiac tissue sections was enlarged after SUV39H1 silencing but reduced after SPP1 downregulation (Figure 6(d)). Likewise, the percentage of the perimeter of the infarcted endocardium was increased by SUV39H1 silencing but reduced by SPP1 silencing (Figure 6(e)). The HE staining also showed that SUV39H1 downregulation aggravated the myocardial injury in rats, and further SPP1 silencing helped alleviate and repair the myocardial injury (Figure 6(f)).

### 3.7. The SUV39H1/SPP1 Axis Modulates Oxidative Stress and Inflammatory Responses

**Figure 7:**
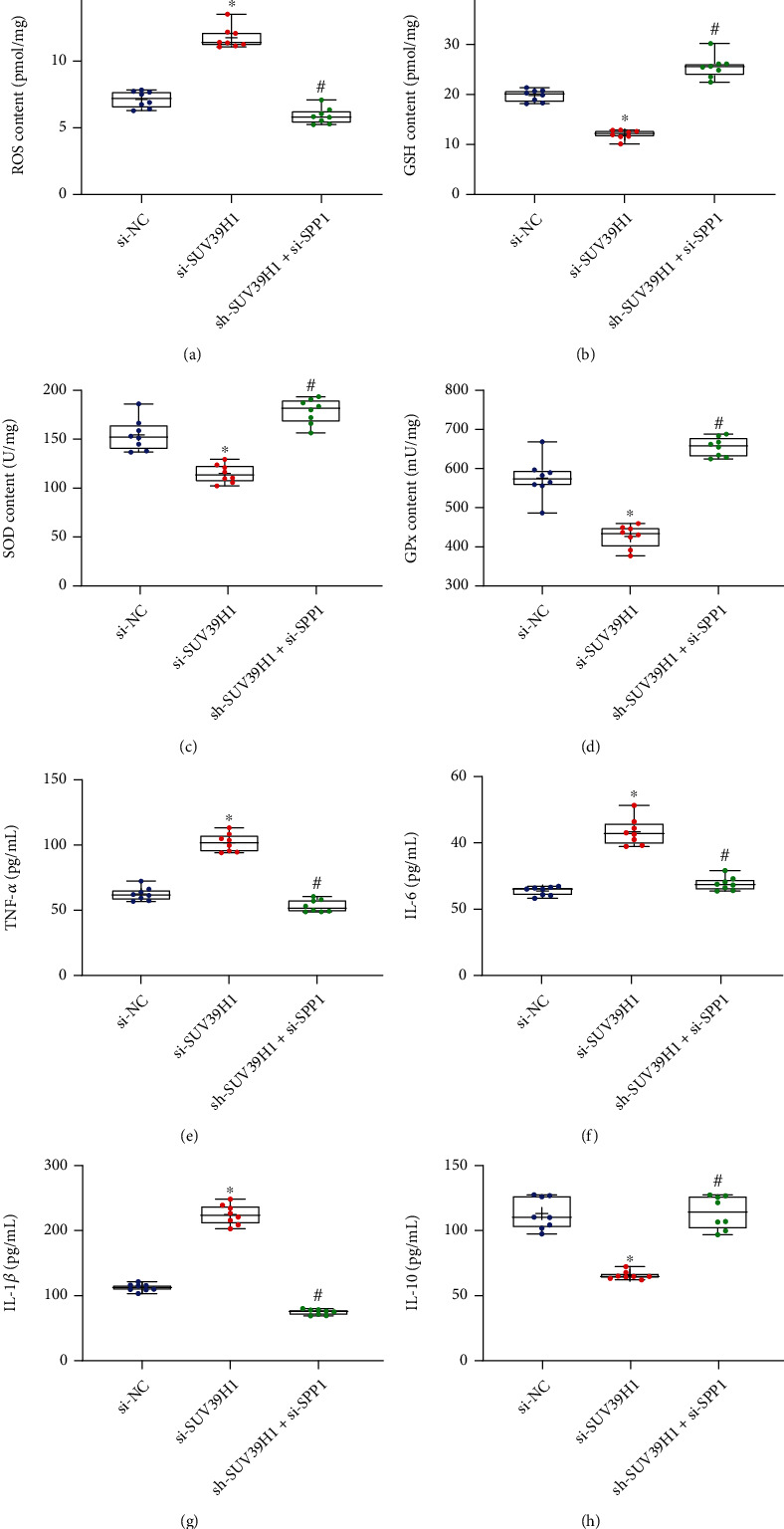
The SUV39H1/SPP1 axis modulates oxidative stress and inflammatory responses. (a–d) Concentration of oxidative stress-related cytokines in rat myocardium examined by the colorimetry (*n* = 8). (e–h) Production of proinflammatory cytokines TNF-*α* (e), IL-6 (f), and IL-1*β* (g) and the anti-inflammatory IL-10 (h) in rat myocardium examined by using ELISA kits (*n* = 8). The si-NC (small interfering RNA-negative control), si-SUV39H1, and si-SUV39H1+si-SPP1 groups refer to the MI model rats treated with si-NC, si-SUV39H1, or si-SPP1. Differences were analyzed by the one-way ANOVA. ^∗^*p* < 0.05 vs. the si-NC group; ^#^*p* < 0.05 vs. the si-SUV39H1 group.

The oxidative stress and inflammatory responses in rats preinjected with si-SUV39H1 and si-SPP1 were explored as well. The concentration of ROS was increased after SUV39H1 inhibition but reduced after SPP1 inhibition (Figure 7(a)). The antioxidants system was damaged after SUV39H1 downregulation but strengthened after SPP1 silencing (Figures 7(b)–7(d)). Moreover, the SUV39H1 silencing promoted the secretion of proinflammatory TNF-*α*, IL-6, and IL-1*β* but reduced the release of anti-inflammatory IL-10 in the cardiac tissues. However, SPP1 silencing led to inverse trends (Figures 7(e)–7(h)).

### 3.8. The SUV39H1/SPP1 Axis Mediates Viability of Cardiomyocytes

**Figure 8:**
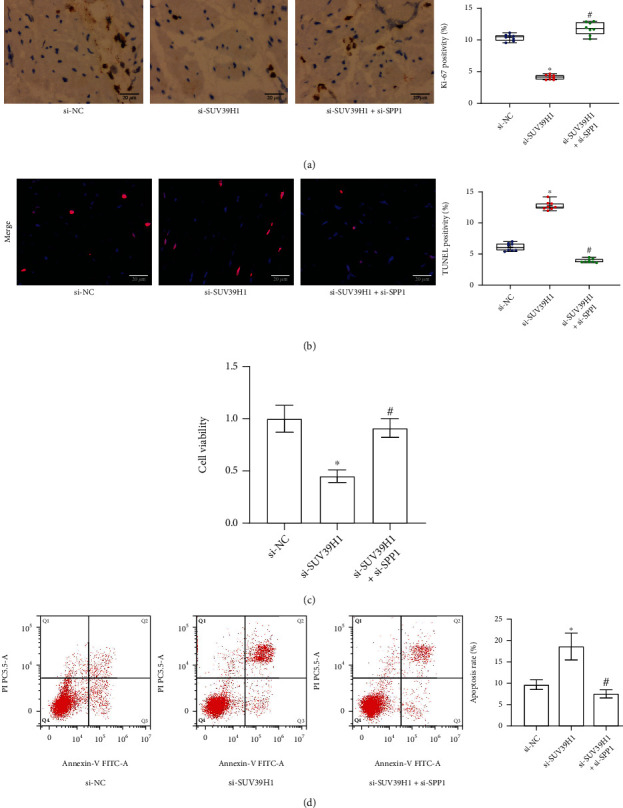
The SUV39H1/SPP1 axis mediates viability of cardiomyocytes. (a) Expression of the proliferation marker Ki-67 in rat cardiac tissues examined by the IHC assay (*n* = 8). (b) Apoptosis of cardiomyocytes in rat cardiac tissues determined by the TUNEL assay (*n* = 8). (c) Viability of H9C2 cells with si-NC, si-SUV39H1, and si-SPP1 transfections examined by the CCK-8 method (*n* = 3). (d) Apoptosis of H9C2 cells with si-NC, si-SUV39H1, and si-SPP1 transfections examined by the flow cytometry (*n* = 3). si-NC (small interfering RNA-negative control), si-SUV39H1, and si-SUV39H1+si-SPP1 groups refer to the MI (myocardial infarction) model rats treated with, or H9C2 cells transfected with si-NC, si-SUV39H1, or si-SPP1. Differences were analyzed by the one-way ANOVA. ^∗^*p* < 0.05 vs. the si-NC group; ^#^*p* < 0.05 vs. the si-SUV39H1 group.

The function of the SUV39H1/SPP1 axis in cardiomyocyte viability was further examined. In the rat myocardium, the expression of Ki-67 was reduced after SUV39H1 silencing but increased after SPP1 downregulation (Figure 8(a)). The TUNEL assay showed that the cell apoptosis rate in rat myocardium was aggravated by SUV39H1 silencing but reduced after SPP1 downregulation (Figure 8(b)). In vitro, the H9C2 cells were transfected with si-SUV39H1 or si-SUV39H1+si-SPP1. The viability of H9C2 cells, according to the CCK-8 method, was suppressed by si-SUV39H1 and increased by si-SPP1 (Figure 8(c)). Moreover, the apoptosis of H9C2 cells was increased following SUV39H1 inhibition but weakened after SPP1 silencing according to the flow cytometry (Figure 8(d)).

## 4. Discussion

The heart attack following MI is a major cause of death around the world [21]. Lcz696 has been demonstrated to be safe and effective and have a superiority over ACEIs in reducing the mortality of patients with HFrEF in the PARADIGM-HF trial [12]. Moreover, lcz696 showed renal protective roles in chronic heart failure [22] and benefits on the cardiac function following MI [23]. In the present study, we report that lcz696 had an array of myocardial protective roles in MI such as alleviating tissue injury, improving cardiac function, and decreasing oxidative stress and inflammatory responses. The epigenetic regulation of SUV39H1 on SPP1 is possibly involved in these events.

In the PIONEER-HF trial, early treatment of lcz696 in patients with MI reduced the level of N-terminal pro-B-type natriuretic peptide and alleviated LV systolic dysfunction [24]. The PARADISE-AMI study also showed that patients with acute MI benefit from the lcz696 treatment [25], and studies are emerging to analyze the treating effect of lcz696 on MI and heart failure [15, 26]. Moreover, several studies reported that the molecular changes are involved in the biofunctions of lcz696. For instance, in a rodent model with MI, lcz696 treatment increased cardiac function and decreased myocardial fibrosis through the downregulation of exosomal microRNA-181a [27]. A recent paper by Shen et al. demonstrated that lcz696 mitigated myocardial injury following MI by suppressing the TAK1/JNK signaling pathway and reducing NLR pyrin family domain containing 3-induced pyroptosis [28]. In this study, a rat model of MI was established. As expected, the lcz696 treatment significantly improved cardiac function and suppressed the heart weight, a major characteristic of the cardiac remodeling [29]. Moreover, the oxidative stress and inflammation and the cardiomyocyte apoptosis in cardiac tissues were suppressed after lcz696 treatment. The oxidative stress induced by excessive ROS is closely correlated with cardiomyocyte injury and inflammation [30, 31], and cardiomyocyte apoptosis can further enlarge infarct size, induce robust inflammatory response, and cardiac injury and remodeling [32]. This body of evidence validated the protective role of lcz696 against MI.

When it comes to the molecules involved, integrated bioinformatic analyzes including gene microarray analysis and the GO functional enrichment analysis revealed that SUV39H1, SPP1, and THBS1 are potential key factors involved in the oxidative stress and inflammation processes in MI. However, the subsequent RT-qPCR suggested the THBS1 expression was not significantly altered, whereas SUV39H1 and SPP1 levels in MI rats were restored to the normal levels by the lcz696 treatment. Moreover, we found that silencing of SUV36H1 in lcz696-treated rats blocked the protective effects of lcz696 and reduced cardiac function and aggravated the myocardial injury. As mentioned previously, artificial upregulation of SUV39H1 effectively reduced infarct size, tissue damage, cardiomyocyte apoptosis, and inflammatory response in diabetic rats of ischemic/reperfusion injury [19]. Recruitment of SUV39H1 to the GATA4 promoter suppressed the GATA4 transcription by triggering H3K9me3 modification, which attenuated hypertrophy and heart failure in mice [33]. Reduced expression of SUV39H1 in visceral fat arteries from obese subjects was correlated with accumulation of ROS and the excessive oxidative stress, leading to obesity-related vascular disease [34]. This body of evidence suggests that SUV39H1 possibly plays a protective role in MI as well. Therefore, it can be opined that upregulation of SUV36H1 is, at least partially, implicated in the protective effects of lcz696. Later, we found that downregulation of SUV39H1 in H9C2 cells led to reduced H3K9me3 level whereas increased SPP1 level in H9C2 cells. Partly in agreement with our findings, SPP1 has been identified as one of the hub genes upregulated in myocardium following MI [16, 35]. The function of SPP1 in cardiac function is not fully elucidated. The rescue experiments in this study suggested that preconditioning of SUV39H1 silencing before lcz696 treatment significantly blocked the myocardium-protective role of lcz696, whereas further preconditioning of SPP1 silencing enhanced cardiac function, suppressed cardiac remodeling, decreased oxidative stress and inflammation, and suppressed cardiomyocyte apoptosis. These results validated that the SUV39H1 upregulation and SPP1 downregulation is involved in the events mediated by lcz696.

**Figure 9:**
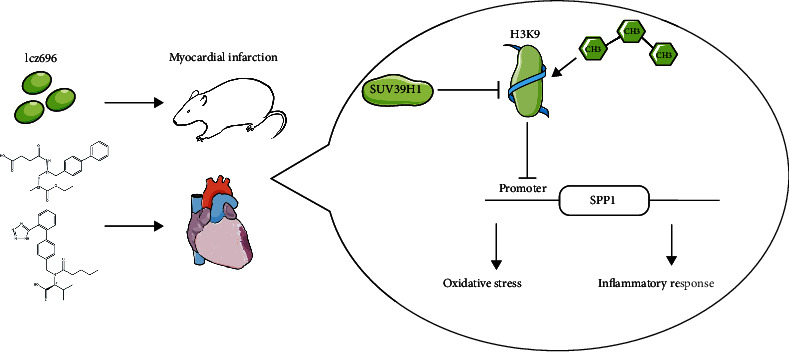
A diagram for molecular mechanism. Lcz696 treatment elevates the level of SUV39H1 in cardiac tissues of rats with MI to suppress SPP1 level through H3K9me3 modification, which suppresses the oxidative and inflammatory response and the related myocardial injury in cardiac tissues.

In conclusion, this study validates the myocardium-protective role of lcz696 and the involvement of SUV39H1-regulated SPP1 suppression in this protection (Figure 9). This study may provide novel ideas to the management of MI using lcz696. One major limitation of the present work is that the exact molecule mechanism by which lcz696 regulates the SUV36H1/SPP1 axis remains unclear. We would like to investigate this issue and explore more molecules responsible for the myocardium-protective effects of lcz696 in the near future.

## Figures and Tables

**Table 1:** Primers for RT-qPCR.

Gene symbol	Orientation	Sequences (5′-3′)
SUV39H1	Forward	TGATGCCAGGCACTTGGTAG
	Reverse	TAAGGGGCCCCAAGTAGGAA

SPP1	Forward	CCAGCCAAGGACCAACTAC
	Reverse	GCTGGCAGTGAAGGACTCAT

THBS1	Forward	TTTGCGGAGAGGACACAGAC
	Reverse	TCATAGTCTTCCTGCCCCGA

GAPDH	Forward	TGTAAGCAATGCCTCCTG
	Reverse	CATCAACGGTCTTCTGTG

Note: RT-qPCR, reverse transcription quantitative polymerase chain reaction; SPP1, secreted phosphoprotein 1; THBS1, thrombospondin 1; GAPDH, glyceraldehyde-3-phosphate dehydrogenase.

**Table 2:** Animal death during the experiments in each group.

Group	Total number	Number of deaths	Death rate
Sham	8	0	0%
MI	12	4	33.33%
lcz696	9	1	11.11%
Si-NC	9	1	11.11%
Si-SUV39H1	12	4	33.33%
Si-SUV39H1+si-SPP1	10	2	20%

Note: MI, myocardial infarction; lcz696, sacubitril valsartan; si-, small-interfering; NC, negative control; SPP1, secreted phosphoprotein 1.

**Table 3:** Hemodynamics of rats in each group (*n* = 8; 1 mmHg = 0.133 kPa).

Group	SBP/mmHg	DBP/mmHg	MAP/mmHg
Sham	141.26 ± 6.07	112.58 ± 6.91	124.79 ± 7.26
MI	113.41 ± 6.25^∗^	76.24 ± 8.52^∗^	94.15 ± 9.33^∗^
lcz696	129.83 ± 7.16^#^	92.15 ± 12.47^#^	110.68 ± 9.54^#^

Note: SBP, systolic blood pressure; DBP, diastolic blood pressure; MAP, mean arterial pressure; MI, myocardial infarction; lcz696, sacubitril valsartan. ^∗^*p* < 0.05 vs. the sham group; ^#^*p* < 0.05 vs. the MI group.

**Table 4:** LV dynamics of rats in each group (*n* = 8; 1 mmHg = 0.133 kPa).

Group	LVSP/mmHg	LVEDP/mmHg	+LVdp/dtmax/	-LVdp/dtmax/
			(mmHg^∗^S^−1^)	(mmHg^∗^S^−1^)
Sham	156.14 ± 15.38	10.36 ± 3.14	9856.28 ± 996.15	−7195.78 ± 1652.74
MI	121.72 ± 8.46^∗^	21.47 ± 3.24^∗^	5924.85 ± 425.71^∗^	−4361.63 ± 567.29^∗^
lcz696	143.69 ± 6.82^#^	13.71 ± 2.05^#^	8542.75 ± 1205.61^#^	−5924.85 ± 1052.35^#^

Note: LV, left ventricular; LVSP, left ventricular systolic pressure; LVEDP, left ventricular end-diastolic pressure; ±LVdp/dtmax, maximum rate of change in LV pressure; MI, myocardial infarction; lcz696, sacubitril valsartan. ^∗^*p* < 0.05 vs. the sham group; ^#^*p* < 0.05 vs. the MI group.

## Data Availability

All the data generated or analyzed during this study are included in this published article.

## Supplementary Materials

Supplementary Materials Supplementary Table 1. A list of the differentially expressed genes.
