# Supplementary material for: Sacubitril Valsartan Enhances Cardiac Function and Alleviates Myocardial Infarction in Rats through a SUV39H1/SPP1 Axis
Source: Oxid Med Cell Longev. 2022 Sep 22;2022:5009289. doi: 10.1155/2022/5009289 (PMC9526637; doi:10.1155/2022/5009289)
Supplement: Supplementary Materials — Supplementary Table 1. A list of the differentially expressed genes. [file 5009289.f1.docx]

**Supplementary Table 1** A list of the differentially expressed genes

| **Down-regulated genes** | **Up-regulated genes** |
| --- | --- |
| SUV39H | FGF7 |
| CYP2E1 | TIMP1 |
| ART1 | FBN1 |
|  | CHI3L1 |
|  | MFAP5 |
|  | HAMP |
|  | LOXL1 |
|  | VCAN |
|  | ANKRD23 |
|  | THBS1 |
|  | FN1 |
|  | LOX |
|  | DKK3 |
|  | FMOD |
|  | MYOT |
|  | CTGF |
|  | WISP2 |
|  | PFKP |
|  | PLA2G2D |
|  | OLR110 |
|  | POSTN |
|  | FCRL2 |
|  | ATP1A3 |
|  | SBSN |
|  | SELPLG |
|  | NCAM1 |
|  | FADS2 |
|  | BGN |
|  | PLOD2 |
|  | NPPA |
|  | LBP |
|  | SPP1 |
|  | C1QA |
|  | CXCL13 |
|  | SLC26A5 |
|  | SFRP2 |
|  | TNFRSF11B |
|  | CTSK |
|  | SFRP1 |
|  | BGLAP |
|  | CLEC3A |
|  | COL12A1 |
|  | COL1A1 |
|  | PTGFR |
|  | RETSAT |
|  | POP5 |
|  | CRLF1 |
|  | COL8A1 |
|  | PTGS2 |
|  | ENC1 |
|  | CDH23 |
|  | LHX4 |
|  | OLR1388 |
|  | KCNB1 |
|  | NLRP6 |
|  | CLEC4A2 |
|  | TGFB2 |
|  | FBLN2 |
|  | F13A1 |
|  | TCEAL7 |
|  | CD55 |
|  | C1QC |
|  | IBSP |
